# Pulmonary tuberculosis among women with cough attending clinics for family planning and maternal and child health in Dar Es Salaam, Tanzania

**DOI:** 10.1186/1471-2458-9-278

**Published:** 2009-08-03

**Authors:** Esther S Ngadaya, Godfrey S Mfinanga, Eliud R Wandwalo, Odd Morkve

**Affiliations:** 1 Centre for International Health, University of Bergen, Bergen, Norway; 2 Muhimbili Medical Research Centre, National Institute for Medical Research, Dar es Salaam, Tanzania; 3 Ministry of Health and Social Welfare, National Tuberculosis and Leprosy Control Programme, (NTLP), P.O. Box 9083 Dar es Salaam, Tanzania; 4 Management Sciences for Health, Dar es Salaam, Tanzania

## Abstract

**Background:**

Tuberculosis (TB) case detection in women has remained low in developing world. This study was conducted to determine the proportion of smear positive TB among women with cough regardless of the duration attending family Planning (FP) and Maternal and child health (MCH) clinics in Dar es Salaam.

**Methods:**

We conducted a cross sectional study in all three municipal hospitals of Dar es Salaam, between October 2007 and June 2008. All women with cough attending FP and MCH clinics were screened for TB by smear microscopy. Pearson chi-square was used to compare group difference for categorical variables. Risk factors for smear positive were estimated by logistics regression with 95% confidence intervals (CI) given for odds ratios indicating statistically significant relationship if the CI did not include one.

**Results:**

We enrolled a total of 749 TB suspects. Five hundred and twenty nine patients (70.6%) were from MCH clinics. Mean (SD) age was 27.6 (5.2) years. A total of 616 (82.2%) patients were coughing for less than two weeks as compared to 133 (17.8%), who coughed for two or more weeks. Among 616 TB suspects, 14 (2.3%) were smear positive TB patients, and of the 133 who had coughed for two or more weeks, 13 (9.8%) were smear positive TB patients. Risk factors associated with smear positive results were having attended more than one visit to any facility prior to diagnosis (OR = 6.8; 95%CI 2.57–18.0) and having HIV/AIDS (OR = 4.4; 95%CI 1.65–11.96). Long duration of cough was not a risk factor for being smear positive (OR = 1.6; 95%CI 0.59–4.49).

**Conclusion:**

The proportion of smear positive TB patients among women with cough attending MCH and FP was 3.8%. Visits to any health facility prior to Diagnosis and HIV infection were risk for having a smear positive TB.

## Background

TB is a problem especially in developing countries. More men than women are diagnosed with TB, whereas more women than men die from TB [1,2].

Since 1983 the annual increase of TB cases in Tanzania has been 2–5% and this is attributed to the increase in HIV/AIDS [3]. Women have been a highly vulnerable group for HIV compared to males, but TB case notification is higher in males as compared to females. Case detection of TB has remained low and it is even lower in women than in men. In 2005, only 37.2% of all smear positive TB patients detected in Tanzania were females [3].

TB case detection in Tanzania is mainly through passive case finding. Passive as oppose to active TB case finding is when symptomatic patients present themselves to the outpatients department (OPD) with cough of two or more weeks with or without accompanying symptoms, are screened for TB [4].

Low TB case detection in women has been associated with socio-cultural factors, low socio-economic status of women and women's tendency of regarding family matters as more important than their own health [5,6]. As shown in a study from India [7], women were found to visit heath facilities for immunization and their children's wellbeing rather than for their own health.

Interventions aimed at integrating passive TB case finding in other clinics like antenatal clinics has proven to be acceptable and has also been recommended in Malawi and South Africa [8,9]. Active case finding for TB revealed a significant number of undiagnosed TB cases among women attending PMTCT clinics in South Africa [9]. However, little is known about the extent of smear positive TB among women with cough attending FP and MCH clinics. This study was therefore, conducted to determine the proportion of smear positive TB among women with cough regardless of their cough duration, attending FP and MCH clinics in Dar es Salaam [3].

## Methods

### Setting

We conducted the study in three health facilities in Dar es Salaam. Dar es Salaam is located in the eastern part of the country, and is administratively divided into three districts namely Kinondoni, Temeke and Ilala, with respective populations of 1,083,913, 768,451 and 634,924 [10]. For operational reasons each district is regarded by the National Tuberculosis and Leprosy Control Program (NTLP) as a region [4]. The facilities included the municipal hospitals of Mwananyamala, Amana and Temeke. We selected Dar es Salaam purposefully because of its high TB burden.

### Study design and data collection

We conducted a cross sectional hospital based study between October 2007 and June 2008. We enrolled all women with cough, attending family planning clinics and those who escorted their children for MCH services. To ensure that all women with cough were enrolled into the study, some of the data collectors were placed at the MCH and FP registration area, in such a way that every woman was asked if she had cough. Those with cough were directed to a study clinician. Those who reported cough, regardless of the duration, were regarded as TB suspects and therefore screened for TB by smear microscopy.

We trained study clinicians and other data collectors from FP and MCH clinics from the selected hospitals. We requested them to register all patients with cough in a study register and asked the patients to submit three sputum samples as per national guidelines[11]. Study registers contained information on patients' socio-demographic characteristics, cough duration in days or weeks and sputum results. Other information included other clinics of consultation for the current respiratory symptoms and number of visits made.

The standard procedure recommended by NTLP in the diagnosis of pulmonary tuberculosis is to examine by smear microscopy all sputum samples from self presenting symptomatic patients [4]. None of the TB case detection activities are routinely conducted at MCH and/or FP clinics. This study was carried out at a time when the Central Tuberculosis Reference Laboratory (CTRL) was conducting quality assurance using Lot Quality Assurance System (LQAS). The results of all laboratories under the study were satisfactory. The quality check for the submitted samples was done according to routine NTLP guidelines [4].

We calculated the minimum sample size of 567 using Epi info version 6.4, statcalc computer software, with the assumption that total population of women aged 15–44 years in Dar es Salaam is 710,486 [10] and we wished to determine with 95% confidence interval (α error of 0.05) a prevalence range of 0.3% to 0.75% of pulmonary tuberculosis (PTB) among women aged 15 to 44 years in Dar es Salaam in 2005 [3].

### Operational definitions

TB suspect: Any woman of reproductive age group with cough, regardless of the duration, who attended FP and MCH clinics.

Smear positive patient: a patient where at least two sputum samples were positive for acid fast bacilli [4].

Smear negative patient: a patient where all three sputum samples were negative for acid fast bacilli [4].

### Ethical considerations

We were granted ethical clearance by the Tanzania Medical Research Coordinating Committee. We obtained informed verbal consent from each interviewee before enrolment into the study. Patients with one smear positive sputum sample were excluded from the analysis but they were referred to the district tuberculosis and leprosy coordinator (DTLC) for treatment and follow up using NTLP procedures. All patients with PTB were also referred to the DTLC for treatment. Non TB patients were treated according to their respective diagnosis.

### Analysis

Data collected were double entered, cleaned and coded using Epi-info version 6 (Centre for Diseased Control and Prevention, Atlanta, GA, USA). We analyzed the data using SPSS version 14 for windows (SPSS Inc, Chicago, IL, USA). The outcome variable was diagnosis of smear positive TB. We calculated the proportion of patients with smear positive TB. We explored possible associations between cough duration and smear results, clinic of diagnosis, place of first presentation and number of visits made prior to diagnosis. We used Pearson chi-Square to compare group difference for categorical variables. Differences were considered statistically significant if p ≤ 5%. Finally, we estimated risk factors for smear positive by logistic regression with 95% (CI) given for odds ratios indicating statistically significant relationship if both values were above or below 1.

## Results

### Baseline profile of the study participants

We enrolled a total of 749 TB suspects. Five hundred and twenty nine patients (70.6%) were from MCH clinics. Table 1 shows the baseline profiles of the 749 study participants according to their smear results. Mean (SD) age was 27.6 (5.2) years (95% CI 27.2–28.0) and median (range) age was 27 (16–50) years. The majority (90.2%) were between 15 to 34 years.

**Table 1:** Risk factors associated with pulmonary TB among women with cough attending FP and MCH clinics.

**Patient characteristics**	**Smear positive TB patients** **n (%)**	**Smear negative patients** **n (%)**	**Total** **n (%)**	**Odds ratio (95%CI)**
**Age distribution**				

15 to 34 yrs	23/27 (85.2)	619/684 (90.5)	642/711 (90.3)	1.66 (0.56–4.94)

> 34 years	4/27 (14.8)	65/684 (9.5)	69/711 (9.7)	

**Marital status**				

Married or cohabiting	17/27 (63.0)	481/686 (70.1)	498/713 (69.8)	1.4 (0.62–3.07)

Single, divorced, or widow	10/27 (37.0)	205/686 (29.9)	215/713 (30.2)	

**Education Level**				

Primary school	24/27 (88.9)	636/686 (92.7)	660/713 (92.6)	1.6 (0.46–5.46)

> primary school	3/27 (11.1)	50/686 (7.3)	45/713 (6.3)	

**Occupation**				

House wife	19/26 (73.1)	374/685 (54.6)	393/711 (55.3)	1.9 (0.85–4.25)

Employed	1/26 (3.8)	19/685 (2.8)	20/711 (2.8)	

Self employed	6/26 (23.1)	292/685 (42.6)	298/711 (41.9)	

Cough duration*				

Two weeks or more	13/27 (48.1)	114. 686 (16.6)	127/713 (17.8)	1.6 (0.59–4.49)

Less than 2 weeks	14/27 (51.9)	572/686 (83.4)	586/713 (82.2)	

**Clinic of attendance**				

MCH	22/27 (81.5)	487/686 (71.0)	509/713 (71.4)	1.8 (0.67–4.81)

FP	5/27 (18.5)	199/686 (29.0)	204/713 (28.6)	

**Place of 1^st ^consultation**				

Government facility	6/22 (27.3)	75/388 (19.3)	81/410 (19.8)	

Private facility	9/22 (40.9)	96/388 (24.7)	105/410 (25.6)	

Pharmacy	7/22 (31.8)	208/388 (53.6)	215/410 (52.4)	

Traditional healer	0	9/388 (2.3)	9/410 (2.2)	

**No of visit to any facility**				

More than one visit	15/21 (71.4)	104/387 (26.9)	119/408 (29.2)	6.8 (95%CI 2.57–18.0)

Only one visit	6/21 (28.6)	283/387 (73.1)	289/408 (70.8)	

**HIV/AIDS self reported**				

HIV/AIDS positive	10/18 (55.6)	43/196 (21.9)	53/214 (24.8)	4.4 (1.65–11.96)

HIV/AIDS negative	8/18 (44.4)	153/196 (78.1)	161/214 (75.2)	

*Cough duration unadjusted for HIV/AIDS: OR 4.7 (95%CI 2.13–10.18)

*Cough duration adjusted for HIV/AIDS: OR = 1.6 (95%CI 0.59–4.49)

Some total do not add up to 749 owing to some missing information.

### Comparison of smear positive PTB patients by cough duration

**Figure 1:**
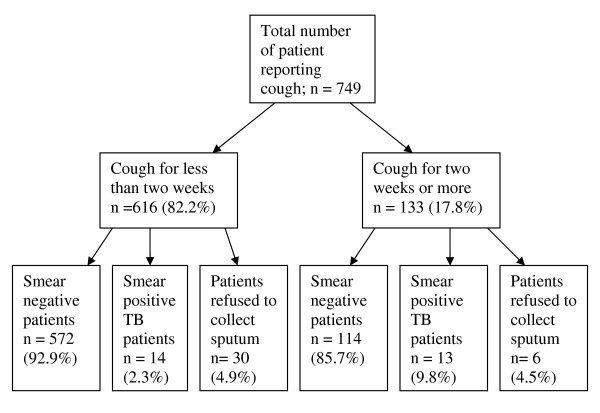
**Comparison of smear results by cough duration**.

A total of 616 (82.2%) patients were coughing for less than two weeks as compared to 133 (17.8%) who coughed for two or more weeks. Among patients who coughed for less that two weeks, 425 (69.0%) were from MCH as compared to only 191 (31.0%) from FP. Among 133 patients who coughed for two or more weeks, 104 (78.2%) were from MCH clinics as compared to 29 (21.8%) from FP. A significantly higher proportion (78.2%) of patients who coughed for two or more weeks attended MCH clinics (X^2 ^= 4.5, p = 0.035). As summarized in figure 1 and table 1, among 616 TB suspects who had coughed for less than two weeks 14 (2.3%) were smear positive TB patients, and of the 133 who had coughed for two or more weeks 13 (9.8%) were smear positive TB patients.

### Comparison between smear positive TB patients and place of first consultation

Among the 749 TB suspects, 430 (57.4%) had visited health facilities for care prior to their diagnosis. Out of these, 124 (28.8%) had coughed for two or more weeks. The most visited facilities were medical stores by 227 (52.4%), government hospitals by 110 (25.6%), private hospitals by 84 (19.5%) and traditional healers by 9 (2.2%) as shown in Table 1. A high proportion (81.5%) of smear positive patients had visited a health facility for care prior to their diagnosis (X^2 ^= 6.6, p = 0.010). It was more common for smear positive patients to have used hospitals as their first point of visit than smear negative patients (*X*^2 ^= 4.4, p = 0.035). Moreover, a higher proportion of smear positive patients (42.9%) made more than two visits prior to diagnosis as compared to smear negative patients (11.4%) (*X*^2 ^= 17.5, p = 0.001).

### Comparison of smear positive PTB patients by clinic

Out of 749 TB suspects, 27 (3.8%) were smear positive TB patients. Among the 27 smear positive patients, 22 (84.6%) were from MCH clinics and 5 (15.4%) were from FP clinics. There was no statistically significant difference when comparing the distribution of proportions of smear positive TB patients among TB suspects from MCH and those from FP clinics (*X*^2 ^= 0.2; p = 0.686).

### Risk factors associated with smear positive results

Risk factors associated with smear positive results were having attended more than one visit to any facility prior to diagnosis (OR = 6.8; 95%CI 2.57–18.0) and having HIV/AIDS (OR = 4.4; 95%CI 1.65–11.96). Long duration of cough, clinic of diagnosis and social demographic characteristics investigated were not risk factors for smear positive TB as shown in Table 1.

## Discussion

The key finding of this study is that the proportion of women with active pulmonary tuberculosis among coughers attending MCH and FP clinics was 3.8%.

According to the existing NTLP guidelines, none of the TB screening activities are done in MCH and FP clinics. Our study indicates that a significant proportion of women with cough attending MCH and FP clinics have pulmonary TB. Taking into consideration the low case detection in women coupled with increase in TB/HIV co-infection, it may be necessary to expand TB diagnostic services to MCH and FP clinics. However, there is a need to conduct more studies to look at the cost-effectiveness and feasibility of expanding TB diagnosis services to the MCH and FP clinics.

Moreover, the majority of the smear positive women were more likely to have visited government hospitals prior to their diagnosis without being recognized as TB suspects. In fact, the majority of them had visited health facilities prior to their diagnosis and made more than one visit but was yet not suspected. Our finding of failure to suspect women is consistent with other studies conducted in Vietnam and Tanzania, where factors like poor knowledge of recognizing and reporting TB symptoms and ignorance among health care workers were associated with delay in TB case detection [11,12].

The majority of women had visited health facilities prior to their diagnosis and made more than one visit but was yet not suspected. This might be explained, though not investigated in this study, by patients' inability to explain well the symptoms and duration of their illness. They could also have first visited health care posts where they were not properly taken care of, e.g. medical stores and traditional healers. Lack of awareness by health personnel and lack of TB diagnostic services could also offer an explanation [6,11,13]. Like in other studies conducted in Brazil and Dar es Salaam, where the probability of having TB did not depend on cough duration [14,15], risk factor for being smear positive TB patient in our study did not depend on the duration of cough. Other risk factors associated with smear positive results were having HIV/AIDS. This is in contrary to other studies where HIV/AIDS positive patients were more likely to be smear negative [16,17]. Though not investigated in this study, but as shown in other studies, possibly the level of immune suppression of our study patients was not so severe to the extent of affecting their TB presentation [18].

Worthy of note also is the fact that women who had a long duration of cough were more likely to be attending MCH than FP clinics. MCH clinics in the study areas were not only the clinics for checking under-fives wellbeing but also acted as referral clinics for the sick children. Studies have indicated that women place the needs of their children and other family activities above their own health. A study in India demonstrated that women tended to visit heath facilities for immunization and their children's wellbeing rather than for their own health [7].

However, it should be kept in mind that the observations from our study are limited to municipal hospitals. A more comprehensive knowledge base could be provided by a multi-site study, with a mixture of governmental and private health facilities, including both urban and rural areas. Another limitation of the study is the potential possibility of imprecise estimates of cough duration, type of facility and number of visits made prior to diagnosis due to recall bias.

## Conclusion

Proportion of smear positive TB patients among women with cough attending MCH and FP is 3.8%. Visits to any facility prior to diagnosis and HIV Co-infection were risk for having a smear positive TB.

## Competing interests

The authors declare that they have no competing interests.

## Authors' contributions

ESN is the primary author who was responsible for conceiving of the research idea, designing of the study, collection of data, analysis and interpretation of the results and writing of the draft and final manuscript. She is also the corresponding author. GSM, ERW and OM participated in proposal write up and were consulted during data collection. Also, they participated during, data analysis and interpretation of the results, writing of the draft and final manuscript. All authors read and approved the final version of the manuscript.

## Pre-publication history

The pre-publication history for this paper can be accessed here:


